# 

**Published:** 1888-12-15

**Authors:** 


CONTENTS.

**Overwork: "A Sad Device of the Devil."** - 161

**Is Saccharin Injurious to Health?** - 162

**The Doctor's Pulpit.**—Present-Day Problems.—Social Parasites - 163

**Glances at the Insane in Other Lands.** By a Medical Superintendent.—II. New Zealand - 164

**Chemists and Their Work** - 165

**The Student's Column.**—Materia Medica - 166

**Physiology and its Uses.**—The Blood-Fluid - 167

**Notes and News** - 168

**Everybody's Page** - 170

**Annotations.**—Bread, or a Stone?—Boycotting Sir Morrell Mackenzie at Edinburgh - 171

**Voyages in Vacation.**—XII. Russian Palaces - 172

**False Impressions.** By Caroline Fothergill, author of "An Enthusiast," "A Voice in the Wilderness," etc. - 173

**Metropolitan Hospitals, Ancient and Modern.**—I - 174

**Editor's Letter-Box**—Women and their Victims - 175

**Scraps and Gleanings** - 176

**Amusements and Relaxation** - 176

EXTRA SUPPLEMENT.—THE NURSING MIRROR.

**En Passant.**—Provincial Items.—Continental Hospitals.—New Restaurant for Women.—Last Week's Conversazione.—Emigration for Women.—Belfast Nurses' Home.—The Power of Parchment - xli

**Grosvenor Gallery Conversazione.—Private Nursing** xlii

**Wages and Washing.**—III. - xliii

**Everybody's Opinion.**—The Pension Fund.—Massage - xliii

**Curious Teething Customs** - xliv

**The Nurses' Bookshelf.**—Nursing Notes - xliv

**Lectures—Vacancies—Notes and Queries** - xliv

